# Changes in Awareness Toward Minor’s Organ Donation Through Structured Information; Survey

**DOI:** 10.3389/ti.2023.10795

**Published:** 2023-02-21

**Authors:** YoungRok Choi, Sanghoon Lee, Yeonhee Lee, Min Hyun Cho, Kyong Ihn, Kyung Chul Yoon, Ji-Man Kang, Seong Heon Kim, Hee Gyung Kang, Nam-Joon Yi

**Affiliations:** ^1^ Department of Surgery, Seoul National University Hospital, Seoul National University College of Medicine, Seoul, Republic of Korea; ^2^ Department of Surgery, Samsung Medical Center, Sungkyunkwan University School of Medicine, Seoul, Republic of Korea; ^3^ Department of Pediatrics, College of Medicine, Seoul St. Mary’s Hospital, The Catholic University of Korea, Seoul, Republic of Korea; ^4^ Department of Pediatrics, School of Medicine, Kyungpook National University, Daegu, Republic of Korea; ^5^ Division of Pediatric Surgery, Department of Surgery, Severance Children’s Hospital, Yonsei University College of Medicine, Seoul, Republic of Korea; ^6^ Department of Surgery, Seoul National University College of Medicine, Seoul National University Boramae Medical Center, Seoul, Republic of Korea; ^7^ Department of Pediatrics, Severance Children’s Hospital, Yonsei University College of Medicine, Seoul, Republic of Korea; ^8^ Department of Pediatrics, Seoul National University Children’s Hospital, Seoul National University College of Medicine, Seoul, Republic of Korea

**Keywords:** living donor liver transplantation, living donor kidney transplantation, minors, long-term complication, informed consent, awareness, organ donation

## Abstract

This study analyzed survey results regarding awareness of living minors’ organ donation. The questionnaires focused on changes in how respondents felt about donations by living minors after eliciting the uncertainty of long-term outcomes for living donors and recipients. The respondents were categorized as minors, adults affiliated with non-medical jobs (Non-Meds), and adults affiliated with medical jobs (Meds). The rates of awareness of living organ donation were significantly different; minors at 86.2%, non-Meds at 82.0%, and Meds at 98.7% (*p* < 0.001). Only 41.4% of Minors and 32.0% of Non-Meds were aware of organ donation by minors, while 70.3% of Meds were (*p* < 0.001). The response rate of opposition to organ donation by minors was highest for Meds and remained the same before and after (54.4%–57.7%, *p* = 0.311). However, the opposition rate in Non-Meds significantly increased (32.4%–46.7%) after learning about the uncertainty of long-term outcomes (*p* = 0.009). The study found that Non-Meds lacked adequate knowledge regarding organ donation by minors and their potential lethal outcomes. Their attitudes toward organ donation by minors could be changed by giving structured information. It is necessary to provide exact information and raise social awareness regarding organ donation by living minors.

## Graphical Abstract

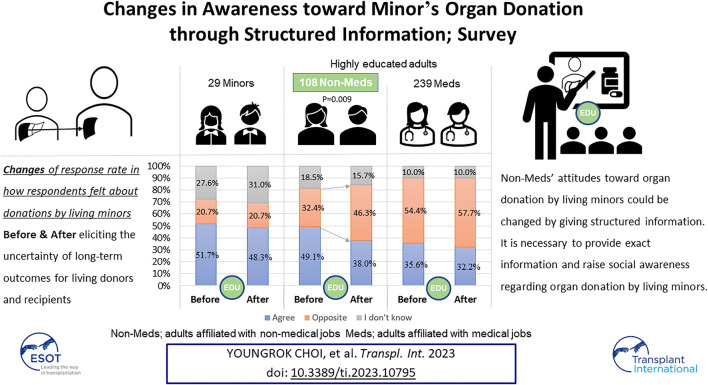

## Introduction

Solid organ transplantation has become a safe and effective treatment option for patients with end-stage renal failure, end-stage hepatic failure, metabolic liver disease, and malignancy. Further, living organ transplantation has been introduced to fill the gap between organ demand and supply, and reduce the high death rate of patients on the transplant waiting list. Living donor organ transplantation is more frequently performed than deceased donor organ transplantation, particularly in Asia and South Korea (1). Nevertheless, organ transplantations have not been performed in sufficient numbers to fulfill the high demand for organ transplants, which is growing every year. As a result, both marginal donors and minors are now legally considered potential organ donors to expand the donor pool (2–6).

**FIGURE 1:**
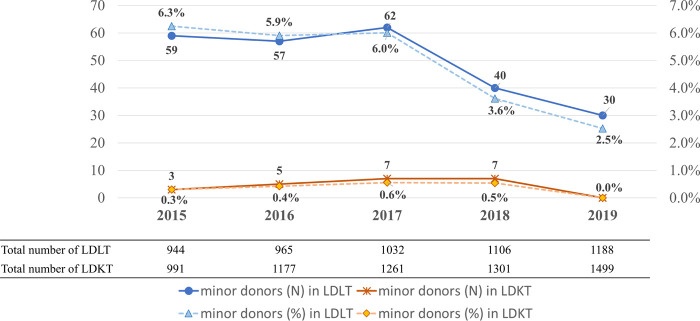
The annual number of living donor transplantations and minor donors in Korea.

The World Health Organization guiding principles on human cells, tissue, and organ transplantation recommend that live organs should not be removed from minors for transplantation. However, several states in the United States and countries such as Canada, Belgium, Luxembourg, Norway, Sweden, the United Kingdom, and Indonesia legally allow the donations of minors under exceptional circumstances (7–12). In South Korea, 30 (2.5%) minors donated their liver in 2019 and 7 (0.5%) minors donated their kidneys in 2018 according to the annual report of the Korean Network for Organ Sharing (Figure 1) (1).

Although the number of minors donating livers and kidneys has decreased in recent years, the practice continues. Minors’ organ donation may be influenced by cultural components that differ from those in Western countries. However, data on the lifelong effects of living donation on live donors as well as minors are lacking. Moreover, the issue of instability, which occurs when minors decide to donate their organs, has to be addressed. Therefore, there is a need to reassess the organ donation of minors (13, 14).

This study aimed to evaluate the knowledge of and attitude toward liver and kidney transplantation (LT and KT) from minor donors in Korea. Moreover, we assessed if receiving structured information on the outcomes of living organ transplantations and donations may change the attitude toward LT and KT from minors.

## Materials and Methods

From June-September 2020, ten professors (five pediatricians and five surgeons) in the Pediatric Committee of the Korea Society of Transplantation created and critically assessed the survey questionnaires and methods. The Institutional Review Board of Seoul National University Hospitalapproved the study protocol (IRB No. 2101-178-1193). Between 1 October and 30 November 2020, the cross-sectional ramdom survey was conducted using a Google form.

The survey link was referred by email to eleven National Universities, ten medical societies, the Korean Bar Association, and three high schools. Data of respondents’ characteristics and responses were collected.

Minors were defined as persons younger than 19 years according to the Korean national regulation. The survey included a structured set of 27 questions. Korean and English-translated versions of the questionnaire were added as Supplementary documents 1 and 2.

**FIGURE 2:**
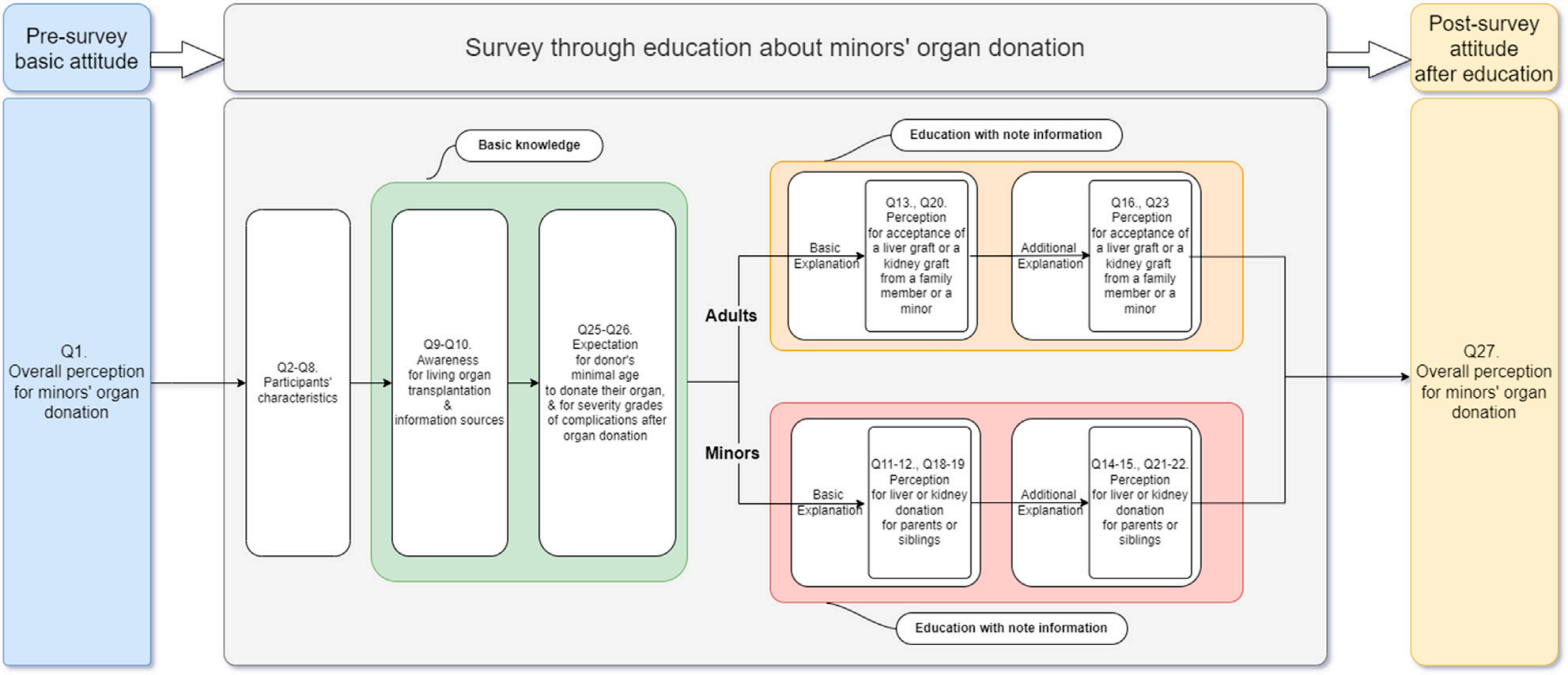
Flowchart of the structured survey for minors’ organ donation.

The questionnaire was divided into three stages, as shown in Figure 2:

(1) Pre-survey stage: Respondents’ basic attitudes (Question 1) toward minors’ organ donation were investigated prior to the main survey.
(2) Survey stage
  (1) Respondents’ characteristics and basic knowledge: The survey stage entails the collection of respondents’ demographic data (Questions 2–8) and investigates their basic knowledge of organ transplantation and minors’ organ donation (Questions 9–10). The purpose of questions 25–26 was to examine respondents’ expectations of the minimum age at which individuals can donate their organs after being made aware of the Korean law governing minors’ organ donation, and the severity grade of donors’ and recipients’ complications following transplantation.
  (2) Respondents’ perception and attitude toward the donation and reception of minors’ organs was further investigated after basic information and additional explanations were provided: The survey in which respondents were educated using structured material was divided into basic and additional explanations. Adults were informed of the overall outcomes and complications associated with the living donation before being asked if they would accept a liver or kidney graft from a family member or minor (Questions 13, 20). Following that, the same questions (Questions 16, 23) were asked after the lack of data, the uncertainty of outcomes associated with living liver donation in minors despite their long-life expectancy, and long-term complications in living kidney donors associated with living with one kidney had been explained to them. Minors among the respondents were also given the same explanations and asked whether they would be willing to donate their liver or kidney to their parents or siblings (Questions 11–12, Questions 18–19, Questions 14–15, and Questions 21–22). Finally, whether providing additional structured explanations influenced respondents’ attitudes was also determined. Questions from 17 to 24 were included to ascertain why respondents altered their decisions after receiving additional explanations.
  (3) Post-survey stage: After the survey stage, Question 27, the same question as Question 1, was asked to investigate whether there was any change in respondents’ attitudes toward minors’ organ transplantation following the questionnaire with additional information.

### Statistical Analysis

Data are mostly presented as numbers and percentages in parenthesis (%), and descriptive statistics summarize the survey data. Answers from respondents were recoreded as categorical variables in the Google form.

Responses to each question were analyzed by categorizing respondents into three groups: minors (Minors), adults affiliated with non-medical jobs (Non-Meds), and adults affiliated with medical jobs (Meds). Pearson’s chi-squared test was used to analyze diffrences between these groups and McNemar’s chi-square test used to analyze diffrences within groups. A *p*-value less than 0.05 were considered statistically significant. The statistical analyses were conducted using SPSS version 25 for Windows (IBM Corporation, Armonk, NY, United States).

## Results

During the study period, 376 people responded. Potenetially eligible respondenst recieved an invitation by email form their institution. All respondenst filled out the questionnaire on a voluntary basis. The basic characteristics of the respondents are detailed in Table 1. There were 347 (92.2%) adults and 29 (7.7%) minors. Of the participants, 202 were males (53.7%) and 174 were females (46.3%). Among minors, 13 were male and 16 were female. Most adults (74.4%) and minors (100%) had 3 or more family members. Of the adults, 332 (95.7%) graduated from college, while 11 (2.9%) finished their education in middle or high school. Eighteen (5.2% of adults) adults and one (3.4% of minors) minor had liver or kidney diseases; among them, more than half had a mild degree of disease severity.

**TABLE 1:** Basic characteristics of the respondents.

		Minors		Adults			
				Non-Meds		Meds	
		N = 29	%	N = 108	%	N = 239	%
Sex	Male	13	44.8	78	72.2	111	46.6
	Female	16	55.2	30	27.8	128	53.6
Age	<19	29	100.0				
	19–29			20	18.5	26	10.9
	30–39			25	23.1	90	37.7
	40–49			54	50	78	32.6
	50–59			8	7.4	37	15.5
	60–69					8	3.3
	>70			1	0.9		
Underlying liver or kidney disease Severity degree of underlying disease	1	1	3.4				
	2			2	1.9	6	50
	3			2	1.9	1	8.3
	4			1	0.9	1	8.3
	5			1	0.9	4	33.3
Educational background		29	100.0				
middle school or High school				7	6.5	4	1.7
University, Graduate school				91	84.3	195	81.6
Post-doctor				9	8.3	37	15.5
Etc.				1	0.9	3	1.3
Family member	1			12	11.1	27	11.3
	2			12	11.1	38	15.9
	3	6	20.7	26	24.1	57	23.8
	4	19	65.5	44	40.7	88	36.8
	>-5	4	13.8	14	13	29	12.1

Of the 347 adults, 239 were Meds; 128 doctors (53.6%), 62 nurses (25.9%), 1 dentist (0.4%), 30 paramedics (12.6%), and 18 medical students (7.5%). Among Meds respondents, 81 (33.9%) were in the surgical field, 26 (10.9%) were in pediatrics, and 23 (9.6%) were in internal medicine.

### Awareness of Organ Transplantation

The awareness of living solid organ donation among the three groups was significantly different; 25 Minors (86.2% of Minors), 89 Non-Meds (82.0% of Non-Meds), and 236 Meds (98.7% of Meds) were aware of living donor organ transplantation (*p* < 0.000). Moreover, as shown in Table 2, only 12 (41.4%) Minors and 34 (32.0%) Non-Meds were aware of minors’ organ donation, whereas 168 (70.3%) Meds were aware of minors’ organ donation (*p <* 0.001).

**TABLE 2:** Awareness regarding minors’ organ donation.

Questions	Minors		Adults				*p-value*
			Non-Meds		Meds		
	N = 29	%	N = 108	%	N = 239	%	
Have you ever heard about living organ transplantation?							
No	4	13.8	19	17.6	3	1.3	*p* < 0.000
Yes	25	86.2	89	82.0	236	98.7	
Do you know that minor can donate their organ?							
No	17	58.6	74	69	71	29.7	*p* < 0.000
Yes		41.4	34	32	168	70.3	

**FIGURE 3:**
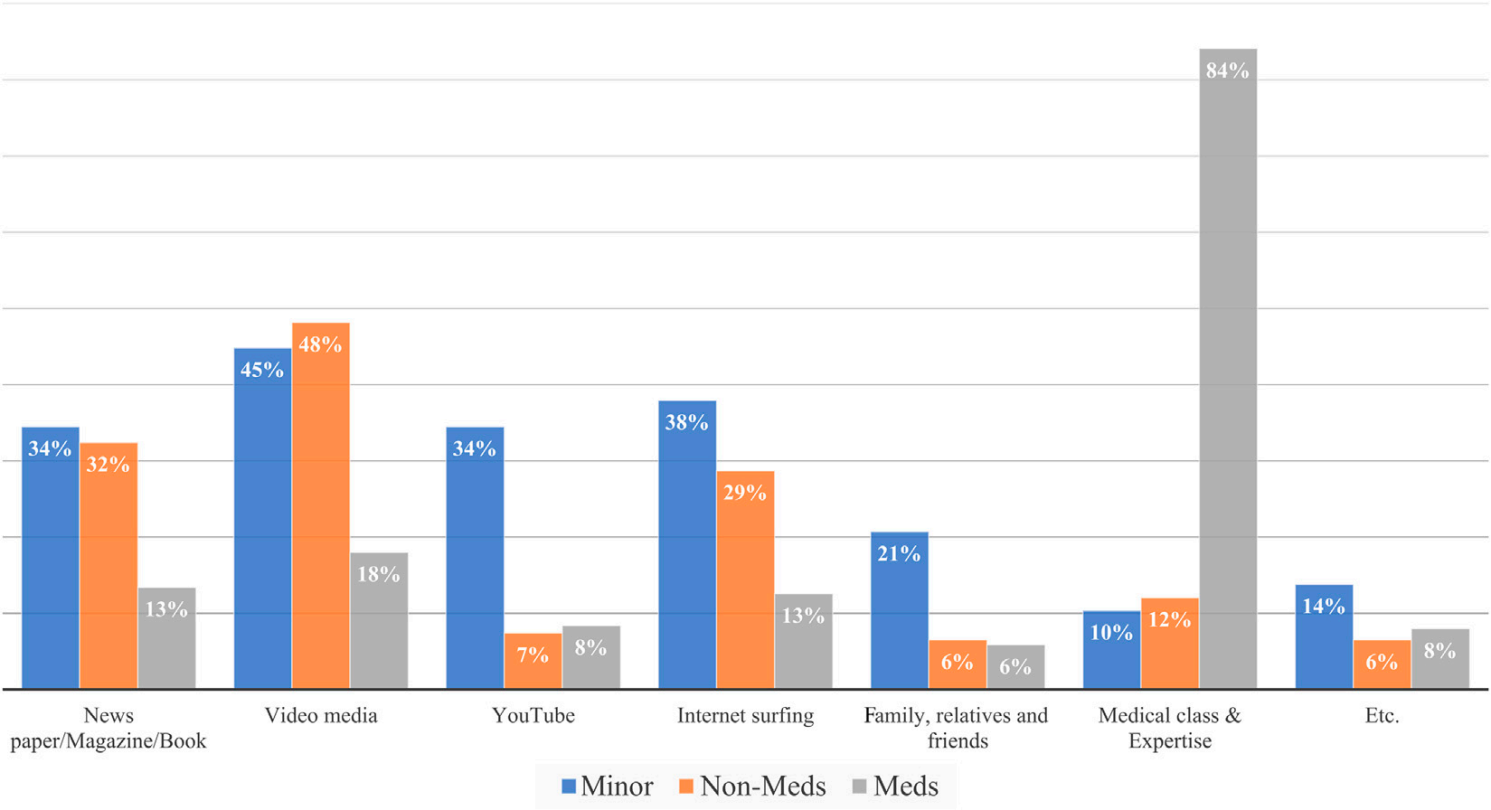
Sources of information on organ transplantation.

Additionally, 26 minors (90%) and 95 Non-Meds (88%) gained knowledge of organ transplantation through various unstructured educational media, while 200 Meds (84%) learned through medical texts and education courses as shown in Figure 3.

### Expected Living Donor’s Age Allowed for Organ Donation by Respondents

**FIGURE 4:**
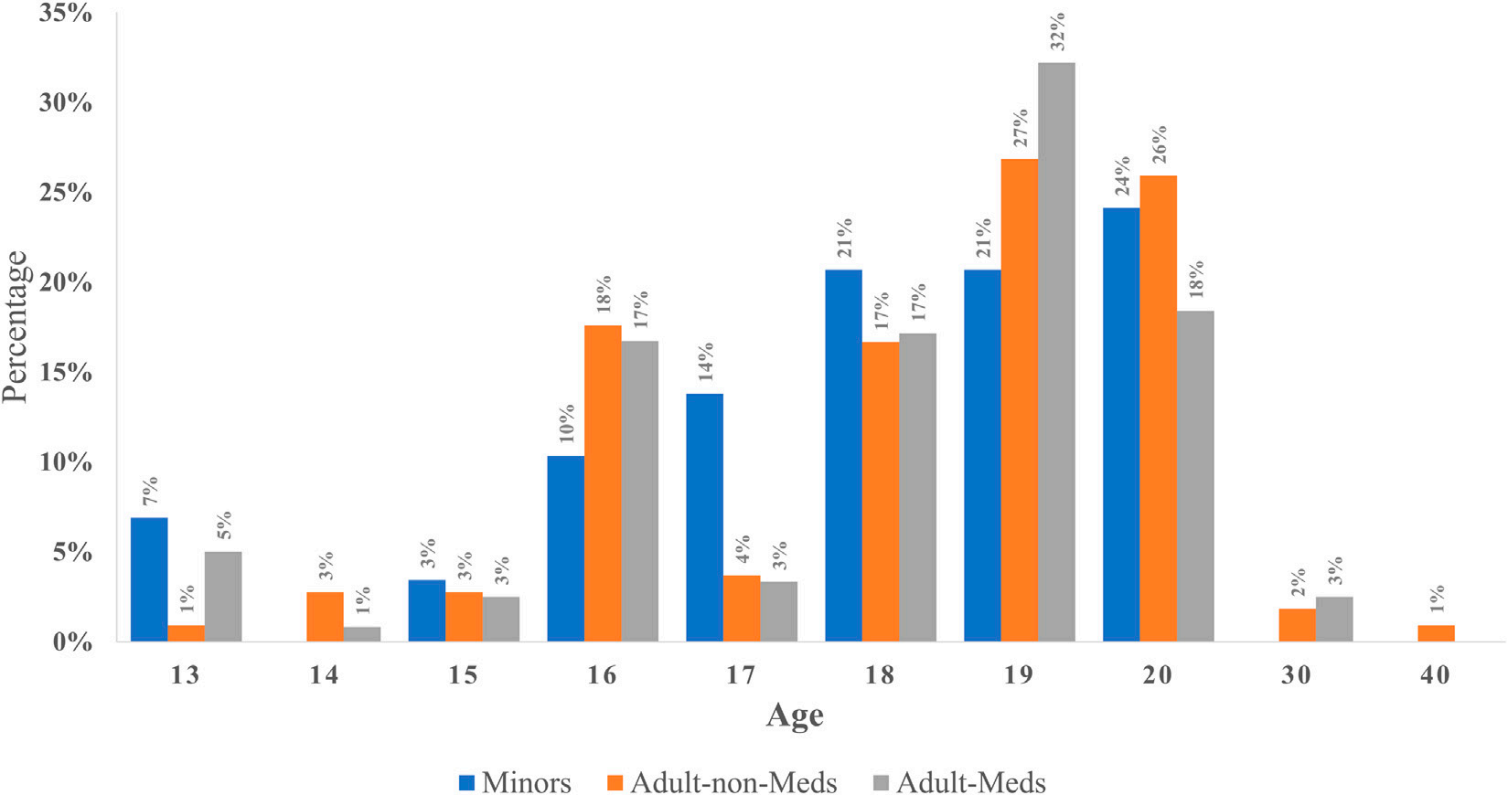
Expected minimum age of a living donor permissible for organ donation among respondents.

There was no difference in respondents’ expectations for the minimum age of a living donor who can donate a solid organ (*p* = 0.561); 19 Minors (65.5%), 100 Non-Meds (72.2%), and 170 Meds (71.1%) expected the minimum age to be 18 or above as shown in Figure 4.

### Awareness of Outcomes for Recipients and Donors After Transplantation

The expected severity grades of complications for living donors after donation were not significantly different among Minors, Non-Meds, and Meds (*p =* 0.707); 16 Minors (55.3%), 61 Non-Meds (56.5%), and 138 Meds (57.7%) thought that living donors might have moderate complications indicating the possibility of death. Meds expected a higher possibility of death for recipients than Minors and Non-Meds (43.9% vs. 27.6%, 20.4%, *p <* 0.0001). Additionally, more Meds (59.0%) expected the possibility of fatal complications in recipients, including intensive care and death, than Minors (34.5%) and Non-Meds (38.9%) did (*p <* 0.0001). Even among Meds, 79.1% did not expect the possibility of living donors’ deaths, although 43.9% of Meds expected the possibility of recipients’ deaths. The details are shown in Table 3.

**TABLE 3:** Expected severity grades of complications for living donors & recipients.

Severity grades of expected complications								
		No complication	Mild complication; medication	Moderate complication; prolonged hospital stay	Severe complications; intensive care	Possibility of death	Total	*p*-value
For Donors	Minors	2 (6.9%)	11 (37.9%)	8 (27.6%)	2 (6.9%)	6 (20.7%)	29 (100%)	*p* = 0.707
	Non-Meds	0	47 (43.5%)	33 (30.6%)	13 (12.0%)	15 (13.9%)	108 (100%)	
	Meds	5 (2.1%)	96 (40.2%)	66 (27.6%)	22 (9.2%)	50 (20.9%)	239 (100%)	
For Recipients	Minors	3 (10.3%)	8 (27.6%)	8 (27.6%)	2 (6.9%)	8 (27.6%)	29 (100%)	*p* < 0.001
	Non-Meds	1 (0.9%)	29 (26.9%)	36 (33.3%)	20 (18.5%)	22 (20.4%)	108 (100%)	
	Meds	0	28 (11.7%)	70 (23.9%)	36 (15.1%)	105 (43.9%)	239 (100%)	

### Under Structured Information, the Changes in the Decision to Donate Their Liver or Kidney in Minors

As shown in Table 4, 96.6% of Minors wanted to donate their liver to their parents after reading basic information. Even after reading additional explanations about the uncertainty of long-term outcomes of living donors, 93.1% of Minors still wanted to donate their liver to their parents (*p =* 0.326). Among Minors, 89.7% wanted to donate their liver to their siblings after receiving a basic explanation and 86.2% after receiving an additional explanation (*p =* 1.000).

**TABLE 4:** Minors’ decision changes regarding donating their organs.

Type of transplantation	Group	To a parent				To a sibling			
		After basic explanation	After additional explanation	∆	*p*-value	After basic explanation	After additional explanation	∆	*p*-value
Liver transplantation	Minors	28 (96.6%)	27 (93.1%)	1 (3.6%)	*p =* 0.326	26 (89.7%)	25 (86.2%)	1 (3.5%)	*p =* 0.100
Kidney transplantation	Minors	28 (96.6%)	27 (93.1%)	1 (3.6%)	*p =* 0.326	26 (89.7%)	25 (86.2%)	1 (3.5%)	*p =* 0.100

A *p-*value less than 0.05 is statistically significant.

Moreover, 96.6% of Minors wanted to donate their kidney to their parents after reading basic information. Even after reading additional explanations about long-term complications associated with the remaining one-sided kidney, 93.1% of Minors still wanted to donate their kidney to their parents (*p =* 0.326); 89.7% of Minors wanted to give their kidney to their siblings after a basic explanation and 86.2% after an additional explanation (*p =* 0.100) (Table 4).

### Under Structured Information, the Changes in the Decision to Reject a Partial Liver or Kidney in Adults

#### Rejection Rate for a Living Liver

As shown in Table 5, 28.5% of all adults chose to reject a partial liver from a family member after reading basic information, and their rejection rate increased to 35.4% after reading additional explanation (*p <* 0.0001). Among all adults, 66.0% decided to reject a partial liver from a minor, although only minors may donate a liver to their family members with a basic explanation. This percentage increased to 72.0% (*p <* 0.0001) after receiving additional explanations about uncertain long-term outcomes.

**TABLE 5:** Adults’ decision changes regarding rejecting living organs.

		Rejection rate a live graft from a family member				Rejection rate a live graft from a minor			
Type of transplantation	Group	After basic explanation	After additional explanation	∆	*p*-value	After basic explanation	After additional explanation	∆	*p*-value
Liver transplantation	All adults	99 (28.5%)	123 (35.4%)	24 (6.9%)	*p <* 0.0001	229 (66.0%)	250 (72.0%)	21 (6.0%)	*p <* 0.0001
	Non-Meds	34 (31.5%)	43 (39.8%)	9 (8.3%)	*p <* 0.006	68 (63.0%)	74 (68.5%)	6 (5.5%)	*p <* 0.014
	Meds	65 (27.2%)	80 (33.5%)	15 (6.3%)	*p <* 0.0001	161 (67A%)	176 (73.6%)	15 (6.2%)	*p <* 0.0001
Kidney transplantation	All adults	102 (29.4%)	134 (38.6%)	32 (9.2%)	*p <* 0.0001	250 (72.0%)	274 (79.0%)	24 (7.0%)	*p <* 0.0001
	Non-Meds	26 (24.1%)	46 (42.6%)	20 (18.5%)	*p <* 0.0001	74 (68.5%)	79 (73.1%)	5 (4.6%)	*p <* 0.0001
	Meds	76 (31.8%)	88 (36.8%)	12 (5.0%)	*p <* 0.0001	176 (73.6%)	195 (81.6%)	19 (8.0%)	*p <* 0.0001

A *p*-value less than 0.05 is statistically significant.

Meds had lower rates to reject a partial liver from a family member than Non-Meds both after basic (27.2% vs. 31.5%) and additional explanation (33.5% vs. 39.8%). However, Meds had higher rates of rejecting a liver from a minor than Non-Meds after basic explanation (67.4% vs. 63.0%, *p <* 0.0001) and after additional explanation about uncertainty (73.6% vs. 68.5%, *p <* 0.0001). The respondents’ rejection rate increased significantly both in Meds (67.4%–73.6%, *p <* 0.0001) and in Non-Meds (63.0%–68.5%, *p <* 0.0014) after additional explanation.

#### Rejection Rate for a Living Kidney

Among adults, 29.4% chose to reject a kidney from a family member after reading basic information, and the rate of rejection increased to 38.6% after recognizing the expected burden on the remnant kidney. Meanwhile, 72.0% of adults rejected receipt of a minor’s kidney. The rejection rate increased to 79.0% after recognizing the expected burden on the remnant kidney.

Meds had higher rejection rates for accepting a kidney from a family member than Non-Meds (31.8% vs. 24.1%) after being given basic information, but Non-Meds had a higher rejection rate than Meds (42.6% vs. 36.8%) after receiving an additional explanation. The change in rejection rate was significant both in Meds (*p <* 0.0001) and in Non-Meds (*p <* 0.0001). Meds had a higher rate of rejecting a kidney from a minor than Non-Meds (73.6% vs. 68.5%, 81.6% vs. 73.1%) after receiving basic and additional explanations. The respondents’ rate for rejecting a minor’s kidney increased from 73.6% to 81.6% significantly in Meds (*p <* 0.0001) and from 68.5% to 73.1% in Non-Meds (*p <* 0.0001) after receiving additional explanations.

#### Changes in Attitude Toward Minors’ Organ Donation

**FIGURE 5:**
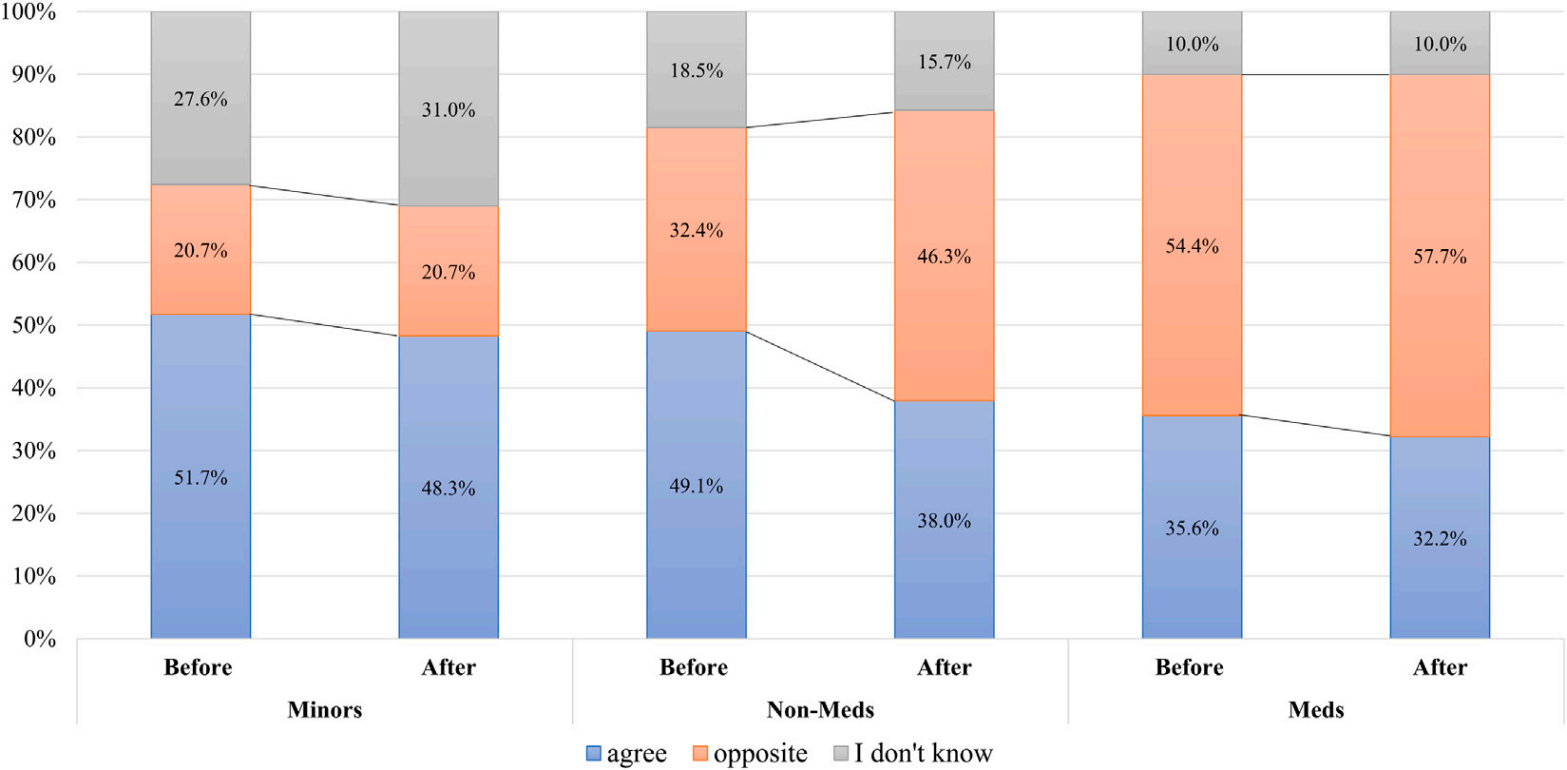
The changes of attitude toward minors’ organ donation.

Among the Minors, 51.7% were willing to donate their organs, 20.7% were reluctant, and 27.6% were indecisive before the survey; and their attitude changed. However, this was not a significant change; 48.3% became willing, 20.7% reluctant, and 31.0% indecisive after providing additional educatonal explanation about long-term outcomes for living donors (*p =* 0.745)*.* All adults had a higher rate of opposing minors’ donations. Among all adults, 39.8% were willing to accept the donation from minors, 47.6% were reluctant, 12.7% were indecisive before the survey; and those opinions significantly changed to 34.0%, 54.2%, and 11.8% respectively with additional explanation (*p =* 0.013). Non-Meds had a higher rate of agreeing to accept minors’ organ donation prior to the survey, but the rate of opposition significantly increased from 32.4% to 46.3% (*p =* 0.009) after they informed about long-term complications. In contrast, the rate of Meds who opposed minors’ donation increased consistently from 54.4% to 57.7% regardless of providing additional information (*p =* 0.311), as shown in Figure 5.

## Discussion

Organ transplantation of both living and deceased donors is practiced worldwide. Nevertheless, supply cannot match the growing demand for organ transplantation. Therefore, to fill the shortage in supply, interest in the vast resource of minors who usually have healthy organs, promising better outcomes of transplantation and better recovery after surgery, became apparent in the field of the living donor organ transplantation (15). However, living organ transplantation using minors as a donor has not been performed in the majority of countries for more than a decade, regardless of the legality of such transplantation.

In Korea, minors above the age of 16 are legally permitted to donate their organs to their family (13). The Korean organ transplantation law stipulates as follows; “Organs, etc. (excluding bone marrow) of a living person who is between 16 and 18 years may not be recovered unless transplanted to a spouse, lineal ascendant, sibling, or relative within the fourth degree.” Further, living minors’ organ donation requires the consent of the donors’ parents. This creates a conflict of interest, as the recipient is frequently the minor’s parent. Korea is one of the countries where living organ transplantation is being performed in more numbers than deceased donor liver transplantation, and organ transplantation using minors’ organs accounts for about 3%, mainly in liver transplantation (1). This trend could be explained by the scarcity of deceased donors in Korea and the family-oriented culture. Nevertheless, this issue has to be considered socially and the law has to be revised, if needed, to prevent unwanted or ill-informed sacrifice from minors for their family, although the outcomes of minors as donors were not poor in several reports from Korea (16–18).

Minors’ judgments may not be conclusive; they are often impulsive and spontaneous when making decisions that may have lasting effects, reflecting a lack of life experience (15). Therefore, it may not be easy for minors to make the right decision regarding organ donation. As can be seen from the results of this study, minors expected the severity of complications for donors and recipients to be less than adults did, which may suggest that minors were not fully aware of the risks and poor prognosis following major surgery. Further, minors’ decisions did not change even after being informed of the uncertainty and poor outcomes after organ transplantation, unlike the adults. Minors are a dependent demographic group, vulnerable to family influence and coercion; they are financially and mentally dependent on their recipients (e.g., parents), which makes it difficult to determine whether their decision to donate is voluntary.

This study found that only 50% of Minors and less than one-third of Non-Meds were aware of living organ transplantation from minors. Further, most of them, 90% of Minors and 88% of Non-Meds picked up the related information from unstructured media and personal communications. This necessitates inviting public attention to the need for structured education on organ transplantation.

Geir et al. (19) reported that the risk of major complications related to living donor nephrectomy is low but represents a potential hazard to the donor. Hong et al. (13) and Choi et al. (13, 14) analyzed the long-term results of living donor liver transplantation using the big data of Korea’s national health insurance and concluded that liver donors have increased long-term mortality risk compared to similar control groups without contraindications to be organ donors, the leading cause of mortality being suicide. The impact of donation on the lives of minors as donors has not been appropriately analyzed, although previous studies using big data showed that physical changes or psychological pain for minors would be significant and that minors may have more difficulty maintaining their mental and physical health (20). Nevertheless, medical personnel (79.1% of Meds) do not expect the possibility of the death of live donors and more than half of Meds (56.1%) do not expect the possibility of the death of recipients. A higher proportion of Minors and Non-Meds expect better outcomes for recipients and donors. Therefore, proper knowledge sharing and education on living donor organ transplantation of both recipients and living donors should be provided to medical experts and non-medical personnel and students. Public attention should be invited to proper education and information on living organ donation. A new living organ donation process should emerge to enable minors to make the right decisions and to protect them from social and familial pressures.

Results of the data on Meds, who received structured information and know the uncertainty of the outcomes of minors’ organ donation, showed differences from those of Non-Meds and Minors. Meds showed consistently higher rates of objection to organ donation by minors than Non-Meds after receiving basic and additional explanations (54.4% vs. 32.4%, 57.7% vs. 46.3% respectively, Figure 5). Pediatricians (65.4%) had the highest rate of opposition among medical professionals, followed by surgeons (64.2%) and medical physicians (56.5%), even though statistically there was no difference (data not shown, *p* = 0.111). Meds tended to accept a liver graft more than a kidney graft from a family member (the rate of rejection of liver graft = 33.5%). They might consider the poor prognosis of a patient with an end-stage liver disease without LT. However, Meds had a strong objection to accepting a liver graft from a minor from their own family (73.6%) (Table 5). Meds’ rejection rates of donation from minors of their own family were higher than their opposition rates of minors’ organ donation from the general population (57.7%).

This study has limitation. First, the small sample sizes of Minors and Non-Meds. Second, his survey was not conducted using a representative sample cohort; rather, it used a random questionnaire. Because there was no financial support for this study, the authors used a Google form and refered the link randomly to 11 National Universities, 10 medical societies, the Korean Bar Association and 3 high schools. The authors planned to collect the answers from 1000 respondants, but only 376 respondents joined the survey for the investigating period.

Nevertheless, the strength of this study is that it is the first survey to investigate the possibility of change in a respondent’s decision about minors’ donation when they are provided with rather unoptimistic information on the reality of LT and KT. Additionally, this survey included minors who expressed their thoughts on minors’ organ donation. Structured education can change the perceptions of non-medical individuals who are in a position to consent to a minor’s organ donation. Non-Meds’ opposition to minors’ organ donation increased after knowing the detailed, uncertain outcomes for organ transplant recipients and living donors. This does not simply indicate a passive conclusion that informed consent on organ donation by minors should include the details on the results of organ donation for minors, but rather, raises fundamental questions about the implementation of organ donation by minors. This will raise awareness toward minors’ organ donation and outcomes for medical experts as well as the general public, not only in Korea but also in many other countries where living organ transplantation is performed.

In conclusion, solid organ donations, including those of minors, and their outcomes for solid organ transplant recipients were not known by non-medical adults or minors. Structured information had the potential to influence adults’ attitudes toward minors’ organ donation. Public attention for proper education and knowledge sharing regarding live organ transplantation and the donation should be addressed to non-medical adults and minors for protecting minors who live under the pressure of living organ donation.

## Data Availability

The raw data supporting the conclusion of this article will be made available by under the permission of the authors and with the approval of the Seoul National University Hospital (SNUH) IRB.

## Abbreviations

KT, kidney transplantation; LT, liver transplantation; n, number; Q, Question.
